# Expression signature of six‐snoRNA serves as novel non‐invasive biomarker for diagnosis and prognosis prediction of renal clear cell carcinoma

**DOI:** 10.1111/jcmm.14886

**Published:** 2020-01-14

**Authors:** Yanyun Zhao, Yuanyuan Yan, Rong Ma, Xuemei Lv, Liwen Zhang, Jinlong Wang, Wenjing Zhu, Lan Zhao, Longyang Jiang, Lin Zhao, Lijie Wen, Bo Yang, Yuzong Chen, Miao He, Mingyan Liu, Minjie Wei

**Affiliations:** ^1^ Department of Pharmacology School of Pharmacy China Medical University Shenyang China; ^2^ Liaoning Engineering Technology Research Center for the Research, Development and Industrialization of Innovative Peptide Drugs China Medical University Shenyang China; ^3^ Urology Department The Second Hospital of Dalian Medical University Dalian China; ^4^ Bioinformatics and Drug Design Group Department of Pharmacy National University of Singapore Singapore City Singapore

**Keywords:** biomarker, clear cell renal cell carcinoma, overall survival, prognostic, recurrence‐free survival, snoRNAs

## Abstract

Increasing evidence has verified that small nucleolar RNAs (snoRNAs) play significant roles in tumorigenesis and exhibit prognostic value in clinical practice. In the study, we analysed the expression profile and clinical relevance of snoRNAs from TCGA database including 530 ccRCC (clear cell renal cell carcinoma) and 72 control cases. By using univariate and multivariate Cox analysis, we established a six‐snoRNA signature and divided patients into high‐risk or low‐risk groups. We found patients in high‐risk group had significantly shorter overall survival and recurrence‐free survival than those in low‐risk group in test series, validation series and entire series by Kaplan‐Meier analysis. We also confirmed this signature had a great accuracy and specificity in 64 clinical tissue cases and 50 serum samples. Then, depending on receiver operating characteristic curve analysis we found the six‐snoRNA signature was an superior indicator better than conventional clinical factors (AUC = 0.732). Furthermore, combining the signature with TNM stage or Fuhrman grade were the optimal indicators (AUC = 0.792; AUC = 0.800) and processed the clinical applied value for ccRCC. Finally, we found the *SNORA70B* and its hose gene *USP34* might directly regulate Wnt signalling pathway to promote tumorigenesis in ccRCC. In general, our study established a six‐snoRNA signature as an independent and superior diagnosis and prognosis indicator for ccRCC.

## 1 INTRODUCTION

Renal cell carcinoma (RCC) is one of the most common malignant tumours in urological malignancies, accounting for about 90% of all adult renal tumours. It is estimated that approximately 73 820 new cases and 14 770 deaths of RCC would occur in the United States during 2019.^1^ ccRCC, accounting for 90% of RCC, represents the most common histologic subtype and aggressive form.^2^ So far, there are mainly two problems in clinical treatment of ccRCC. On the one hand, it is difficult to diagnose ccRCC early, especially for patients with small renal masses (pT1a, ≤4 cm).^3^ On the other hand, there are no specific prognostic indicators for predicting overall survival (OS) and recurrence‐free survival (RFS) of ccRCC patients largely depended on stage, grade, tumour size and so on.^4^ Although some biomarkers, such as *VHL*,^5^ *BAP1* 6 mutations and overexpression of *AHNAK2* 7 and *SLC6A3*,^8^ have been discovered, few markers have been validated their diagnostic or predictive power in clinical practice.^9^ Therefore, it is imperative to identify more sensitive and reliable biomarker for ccRCC.

Small nucleolar RNAs (snoRNAs) are a kind of non‐coding RNA with 60‐300 nucleotides in length,^10^ involving in guiding site‐specific post‐transcriptional modification of rRNAs, tRNAs, snoRNAs and snRNAs.^11^ Small nucleolar RNAs are primarily classified into H/ACA box and C/D box snoRNAs based on their structure and main function. H/ACA box snoRNAs guide pseudouridylation of nucleotides, whereas C/D box is responsible for 2′‐O‐methylation.^11^, ^12^, ^13^ The defects in ribosome maturation and function can destroy important protein synthesis processes and lead to diseases, especially cancer.^14^ In recent years, new and previously unrecognized functions of snoRNAs have been discovered in various cancers, revealing that the snoRNAs might associate with tumorigenesis. *SNORA42*,^15^ *SNORD33*, *SNORD66*, *SNORD76*,^16^ *SNORD78* 17 and *SNORD114.1* 18 have been reported the potential prognostic value in colorectal cancer, non–small‐cell lung cancer and peripheral artery disease. Furthermore, Gong et al^19^ analysed snoRNA expression landscape across 31 cancer types and observed that overexpression of several snoRNAs in kidney renal clear cell carcinoma (KIRC), suggesting snoRNAs may play important roles in KIRC.

Despite the emerging knowledge about the role of snoRNAs in cancer, the clinical relevance of snoRNAs in ccRCC has not been investigated systematically. In this study, we identified a six‐snoRNA signature as an independent and specific predictor to predict the prognosis of ccRCC patients from TCGA database and validated its clinical application value in a subset of ccRCC tissue and serum by qRT‐PCR. Hence, the six‐snoRNA signature might provide a prospective prognostic biomarker set and potential therapeutic targets for ccRCC.

## 2 MATERIAL AND METHODS

### 2.1 ccRCC datasets preparation

The TCGA ccRCC tumour and paired adjacent tissue samples RNA‐seq gene expression data (HTSeq‐Counts) and corresponding clinical data were downloaded from The Cancer Genome Atlas of the National Cancer Institute (TCGA, http://cancergenome.nih.gov). After removal of the nine samples without survival study and clinical information, a total of 530 ccRCC patients and 72 control cases were analysed in the present study. The downloaded clinical data included age, gender, TNM stage, Fuhrman grade, haemoglobin levels and so on for ccRCC. Simultaneously, we also downloaded RNA‐seq gene expression data (HTSeq‐Counts) and corresponding clinical data for patients with papillary renal cell carcinoma (KIRP, 281 patients and 32 control cases included), chromophobe renal cell carcinoma (KICH, 65 patients and 24 control cases included) and bladder cancer (411 patients and 18 control cases included).

### 2.2 Gene selection and gene signature building

First, the differentially expressed snoRNAs were screened using ‘edgeR’ package. Then, the 530 ccRCC cases in TCGA data sets were randomly assigned into test series (N = 371) and internal validation series (N = 159) at ratio 7:3 (Table S1). By univariable and multivariable Cox regression analysis, we established a prognostic signature and validated it in the internal validation series and entire validation series. A snoRNA‐based risk score model formula was conducted in the test series as follows:

$$\text{snoRNA}\;-\;\text{based}\;\text{resk}\;\text{score}=\sum_{i=1}^{n}{\text{Coe}}_{i}\times {\text{EV}}_{i},$$

where n was the number of predicted snoRNAs, Coe_i_ indicated the coefficient of the *i*th snoRNA in multivariable Cox regression analysis, and EV_i_ represented the expression value of the *i*th snoRNA. The snoRNAs with Coe*_i_* < 0 were considered as protective factors, whereas those with Coe*_i_* > 0 were considered as risky factors.

### 2.3 Patients and clinical specimens

We recruited 32 pairs of matched fresh‐frozen ccRCC and adjacent normal tissue, and also serum from 25 cases patients with ccRCC and 25 control cases. The tissue samples and corresponding clinical pathology data were from the Second Hospital of Dalian Medical University, and the serum samples and corresponding clinical pathology data were from the Second and Fourth Affiliated Hospitals of China Medical University. The study was approved by Institutional Review Board.

### 2.4 RNA isolation

The recruited serum sample was allowed to coagulate at room temperature for 30 min and then centrifuged at 1000 g for 10 min to take the supernatant. The supernatant was centrifuged again for 12 000 g for 15 min to remove all cellular components and immediately stored at −80°C. For tissue and serum RNA isolation, 1mL TRIzol (Invitrogen) was added to 50 mg of tissue or 200 μL of serum and total RNA extraction according to the manufacturer's instructions.^20^ Purified RNA was quantified using NanoDrop 2000 (Thermo Scientific). In general, the yield was 0.3‐1 μg/mg tissue or 0.1‐0.5 ng RNA/mL serum.

### 2.5 qRT‐PCR

Reverse transcription was performed from 500 ng of total RNA using the ReverTra Ace qPCR RT Kit. 37°C for 15 min, followed by reverse transcriptase inactivation at 85°C for 5 min was utilized. The cDNA was used for PCR or stored at −80°C immediately. The expression of snoRNAs was analysed by custom TaqMan assays (Applied Biosystems), using the QuantStudio™ 3 Flex Real‐Time PCR System (Applied Biosystems) and using the following condition: 95°C for 1 min, followed by 40 cycles of 95°C for 15 s, 60°C for 30 s and 72°C for 1 min. Primers were as follows: *SNORA2* forward (5′‐ATTCAAGGCCAGCAGTTTGC‐3′) and *SNORA2* reverse (5′‐AGATGGCCAACAGACCATGAA‐3′); *SNORD12B* forward (5′‐TCCTGCTGGCATATATGATGACTT‐3′) and *SNORD12B* reverse (5′‐GCTCAAGCTGGCATATCAGAC‐3′); *SNORA59B* forward (5′‐CCTCACAACGTTTGTGCCTC‐3′) and *SNORA59B* reverse (5′‐AGCTGTTCCTTATCACCAACGA‐3′); *SNORA70B* forward (5′‐TCCTTATGGGGGTCCAGTGT‐3′) and *SNORA70B* reverse (5′‐CAACAAACAGGCCGCATACA‐3′); *SNORD93* forward (5′‐GCCAAGGATGAGAACTCTAATCTGA‐3′) and *SNORD93* reverse (5′‐GGCCTCAGGTAAATCCTTTAATCCA‐3′); *SNORD116‐2* forward (5′‐TGGATCGATGATGAGTCCCC‐3′) and *SNORD116‐2* reverse (5′‐AGTTCCGATGAGAATGACGGT‐3′). The expression levels of snoRNAs were calculated using the 2^−Δ^ *^Ct^* method.^21^

### 2.6 Principle component analysis

Principle component analysis (PCA; 3‐D PCA plots were generated using SPSS 22.0) was performed and visualized to compare variations of within‐ and between‐sample groups. The expression profiles of snoRNAs were normalized so that the data approach normal distributions.

### 2.7 GO term and KEGG enrichment analysis

The clusterProfiler package was implemented to further explore the biological function of snoRNAs including biological process (BP) and Kyoto Encyclopedia of Genes and Genomes (KEGG) analysis.^22^ *P* < .05 was considered a significant enrichment.

### 2.8 Construction of nomogram predictive model

The ‘rms’, ‘nomogramEx’ and ‘regplot’ R package were used to construct nomogram. The nomogram was used to predict the survival rate of ccRCC with multiple indicators.^23^ The total points were obtained by plus the points of each prognostic parameters, and patients with higher total points had worse survival. Separating capacity of the nomogram was tested by Harrell's concordance index (C‐index).

### 2.9 Statistical analysis

Overall survival differences between patients in high‐risk and low‐risk groups were estimated by Kaplan‐Meier survival curve and calculated using the log‐rank test. The receiver operating characteristic (ROC) curve was made to determine the sensitivity and specificity of the snoRNA signature through calculating the area under curve (AUC). The Cancer Cell Line Encyclopedia (CCLE) data were downloaded to explore the expression of snoRNAs (http://www.broadinstitute.org/ccle).^24^ The GEPIA (http://gepia.cancer-pku.cn) website was used to analyse expression correlation between two genes.^25^ We compared two groups using *t* test for numerical variables, one‐way ANOVA for different groups and chi‐square test or Fisher's exact test for categorical variables. The SNORic (http://bioinfo.life.hust.edu.cn/SNORic) was used to examine the correlation between snoRNAs and copy number variation (CNV), DNA methylation and protein expression. The methsurv was used to evaluate prognostic properties of DNA methylation (https://biit.cs.ut.ee/methsurv). All statistical analyses were carried out using R (https://www.r-project.org/, v3.5.1), SPSS 22.0 (SPSS Inc) and GraphPad Prism7 (GraphPad Software Inc.).

## 3 RESULTS

### 3.1 Identifying a six‐snoRNA signature as a potential prognostic marker for ccRCC

**Figure 1:**
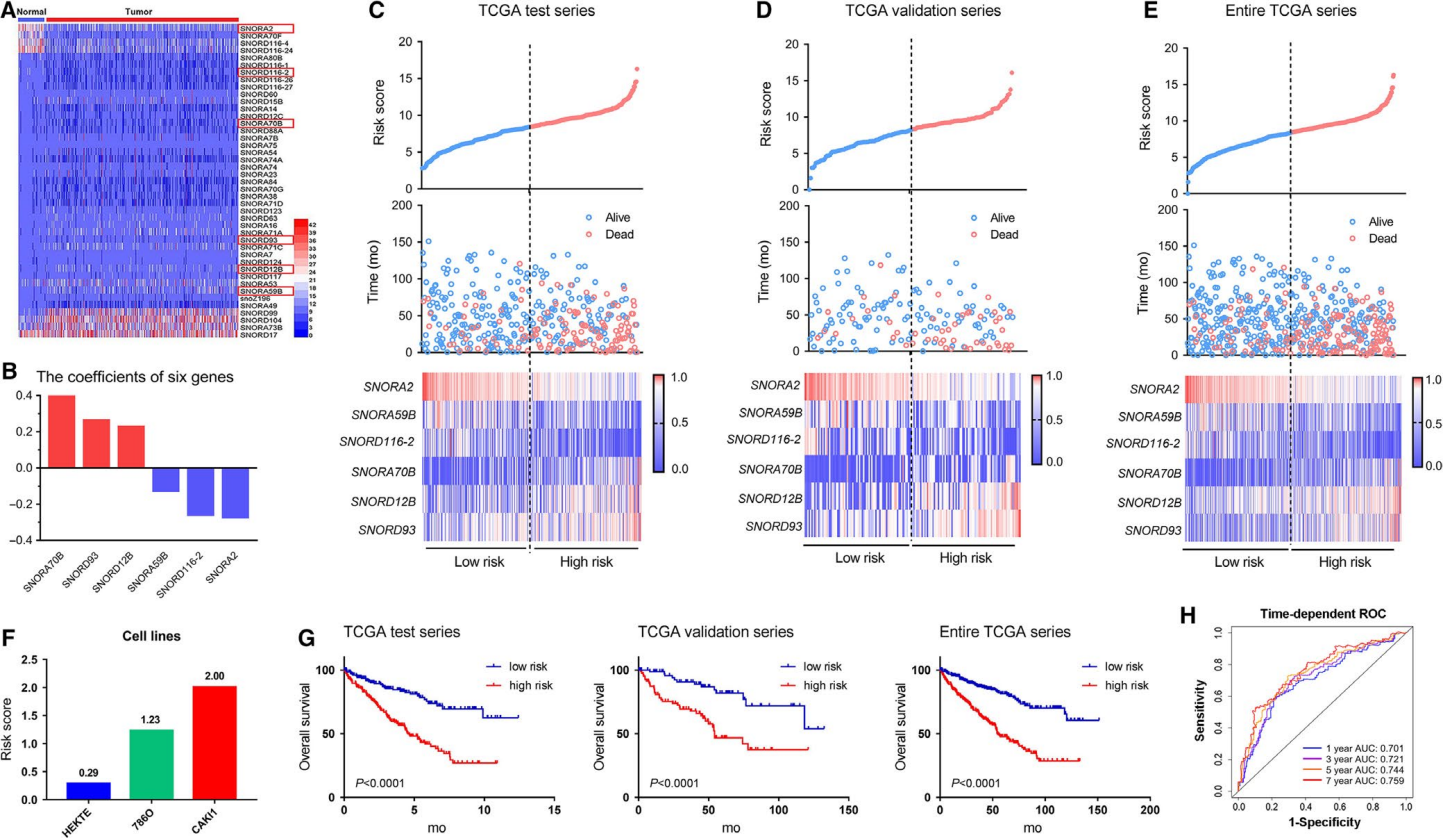
Six ccRCC relevant snoRNAs were identified. A, The differentially expressed snoRNA status in TCGA data between ccRCC tissue and adjacent tissue (N = 530). B, The coefficients of six snoRNAs. Risk score distribution patients’ survival time and status and heat map of six‐snoRNA expression in test series (C), validation series (D) and entire series (E). Rows represent snoRNAs, and columns represent patients. F, The risk score of cell lines including HEKTE (normal renal cells), 786‐O ccRCC cell line (primary) and Caki‐1 ccRCC cell line (metastatic). G, Kaplan‐Meier analysis for OS of the six‐snoRNA signature in test series, validation series and entire series. H, The time‐dependent ROC analysis of the sensitivity and specificity of the signature in entire series. The differences between the two curves were determined by the two‐side log‐rank test

Although some small nucleolar RNAs (snoRNAs) had prognostic value in various cancer,^15^, ^16^ the expression level and clinical significance of snoRNAs in ccRCC have not been established systematically. Therefore, we first determined the expression level of snoRNAs by analysing TCGA database and identified 43 significantly differential expression snoRNAs between 530 ccRCC cases and 72 control cases (|Log FC|>1, *P* < .05, Figure 1A and Table S2). Then, to evaluate prognostic value of snoRNAs in ccRCC, we subjected the 43 snoRNA expression data of the test series to univariable Cox analysis and screened 15 snoRNAs which were significantly associated with prognosis of ccRCC patients (Table S3). To further determine whether those snoRNAs could be independent risk factors for predicting survival of ccRCC patients, those 15 candidate snoRNAs were further analysed by multivariable Cox analysis in the test series. Finally, six snoRNAs (*P* < .05) were identified as the independent risk factors markedly related to survival, including *SNORA2*, *SNORD12B*, *SNORA59B*, *SNORA70B*, *SNORD93* and *SNORD116‐2* (Table S4). To better predict the prognosis of ccRCC patients, these six snoRNAs were further used to build a predictive snoRNA signature. The coefficients were depended on the HR of each snoRNA calculated by multivariable Cox regression in the test series (Figure 1B). We established the risk score formula as follows: risk score = (−0.2791*SNORA2) + (−0.2461*SNORD116‐2) + (−0.1322*SNORA59B) + (0.2680*SNORD93) + (0.2330*SNORD12B) + (0.4199*SNORA70B). To investigate the effect of risk score on survival status of patients and expression level of each snoRNA, we then calculated risk score for each patient based on the risk score formula in the test series, and ranked them according to their scores. The distribution of risk score, the survival status of the ccRCC patients and these snoRNA expression profiles were also obtained. In the test series (Figure 1C), we found that patients in high‐risk group tended to express high level of risk snoRNAs (*SNORD12B*, *SNORA70B* and *SNORD93*), whereas patients in low‐risk group tended to express high level of protective snoRNAs (*SNORA2*, *SNORA59B* and *SNORD116‐2*). Similar results were also observed in validation series and entire TCGA series (Figure 1D‐E). To further explore the validity of this signature, we analysed the risk score in ccRCC cell lines and the result showed that risk score of HEKTE, 786‐O and Caki‐1 cell lines was 0.29, 1.23 and 2.00, respectively. We found the risk score of tumour cells (786‐O and Caki‐1) was higher than that of normal cells (HEKTE), which implied that our six‐snoRNA signature had a good diagnostic value to distinguish tumour from non‐tumour cell lines. Furthermore, the risk score of highly malignant cells (Caki‐1) was higher than that of lowly malignant cells (786‐O), which implied that our six‐snoRNA signature had a good prognostic value for ccRCC (Figure 1F).

To evaluate the prognostic utility of the six‐snoRNA signature in ccRCC, patients were divided into high‐risk group or low‐risk group by the median risk score as the cut‐off point. By Kaplan‐Meier analysis, we found patients in high‐risk group had significantly shorter OS than those in low‐risk group (*P* < .0001) (Figure 1G). Besides, similar results were also observed in validation series and entire TCGA series (Figure 1G). Furthermore, we also analysed the RFS and found patients in high‐risk group had significantly shorter RFS than those in low‐risk group in test series (*P* < .0001), validation series (*P* = .0071) and entire series (*P* < .0001) (Figure S1A) What is more, patients with recurrence had higher risk score than those without recurrence (Figure S1B).

To explore the sensitivity and specificity of the six‐snoRNA signature, we conducted time‐dependent ROC analysis and result showed that the prognostic accuracy of the signature was 0.701, 0.721, 0.744 and 0.759 for 1, 3, 5 and 7 years in entire series which increased with time prolonging (Figure 1H). Taken together, this six‐snoRNA signature is a potentially helpful biomarker for predicting OS and RFS of ccRCC patients.

### 3.2 High‐risk score is associated with advanced TNM, higher Fuhrman grade and low haemoglobin level

**Figure 2:**
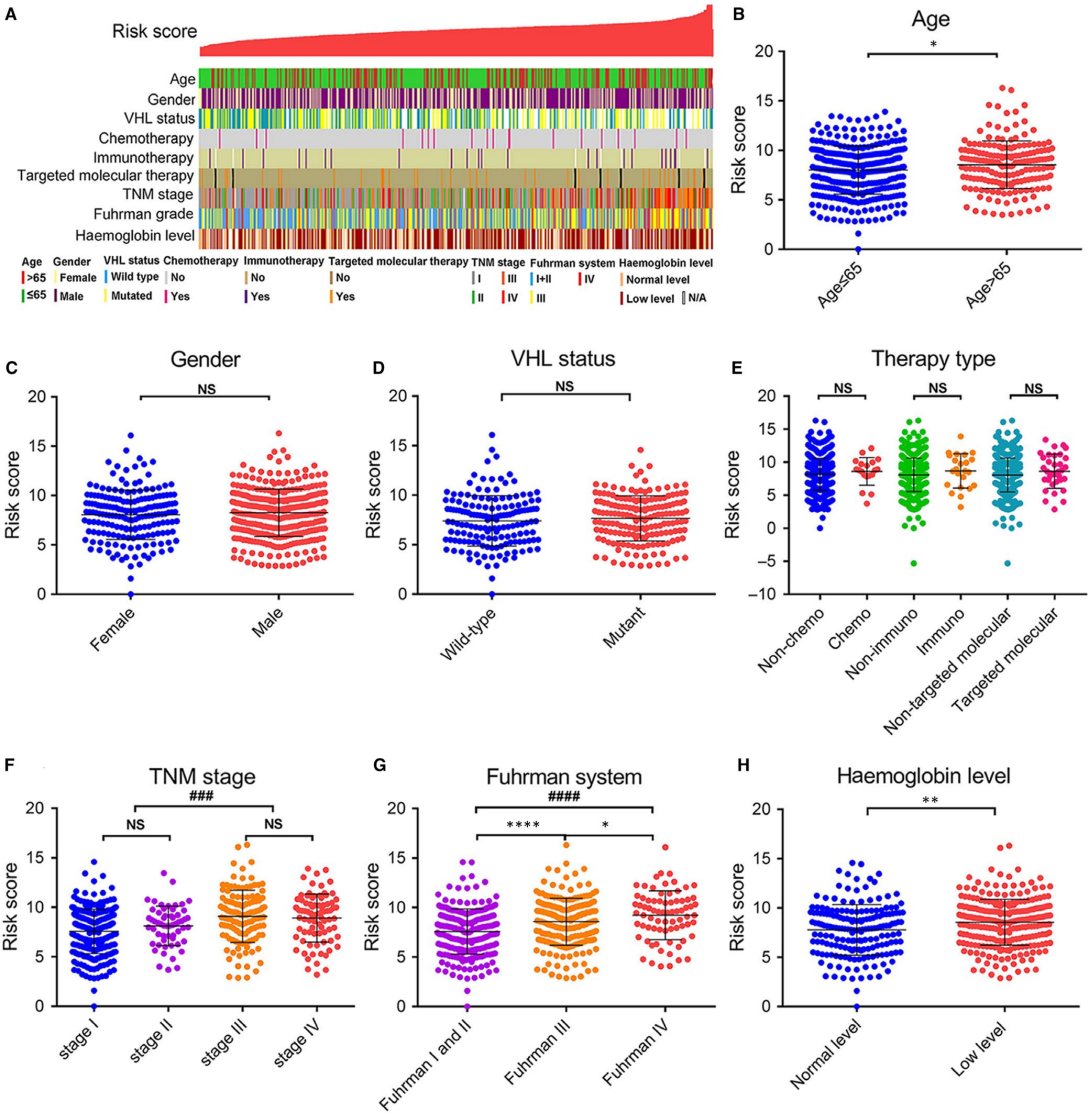
Relationship between the predictive signature risk score and clinicopathologic characteristics. A, The clinicopathologic information of patients in TCGA database, arranged by the increasing risk score. The distribution of risk score in patients stratified by age (B), gender (C), VHL status (D), therapy type (E), TNM stage (F), Fuhrman grade (G) and haemoglobin level (H). *P values were measured by unpaired *t* test. *P < .05, **P < .01, ***P < .001, ****P < .0001. #, one‐way ANOVA for different pathological stages. ###*P* < .001, ####*P* < .0001

To further comprehensively investigate whether there was a relationship between the risk score and pathological characteristics, patients were arranged according to their risk score. The results showed obviously asymmetric distribution of the Fuhrman grade, TNM stage and haemoglobin level (Figure 2A). We found elevated risk score was positively associated with advanced TNM stage, higher Fuhrman grade and lower haemoglobin level. However, age, gender, VHL status, chemotherapy, immunotherapy and target molecular therapy showed no difference in the distribution (Figure 2A). We further compared the risk score of patients separated by clinical characteristics. Some clinical characteristics were not associated with our risk scores, such as gender status, VHL status and therapy type (Figure 2C‐E). However, the risk score was highly related to age, TNM stage, Fuhrman grade and haemoglobin level (Figure 2B, 2‐H). To illustrate, the risk score was higher in TNM stages III and IV, compared with TNM stages I and II (Figure 2F). Contrasted to the Fuhrman I and II stages, the risk score was higher in Fuhrman III and Fuhrman IV stages (Figure 2G). Furthermore, the risk score was higher in low haemoglobin level (Figure 2H). We also found that along with the ccRCC stage developed, the risk score was higher (Figure 2F,2). These results demonstrated that elevated risk score indicated advanced TNM stage, higher Fuhrman grade and lower haemoglobin level.

### 3.3 Prognostic value of the six‐snoRNA signature is independent of conventional clinical factors

To further appraise the predictive effect of the six‐snoRNA signature and other clinicopathologic characteristics on survival status, we performed univariable and multivariable Cox analysis to determine whether the six‐snoRNA signature could be an independent risk factor for evaluating prognosis of ccRCC patients. Univariable Cox analysis revealed that age, the six‐snoRNA signature, TNM stage, Fuhrman grade and haemoglobin level were significantly related to the patients' survival status (Table S5). Multivariable Cox regression showed that the six‐snoRNA signature, age, TNM stage, Fuhrman grade and haemoglobin were independent prognostic factors (Table S6). In addition, the six‐snoRNA signature was also an independent risk factor for RFS by univariable and multivariable Cox regression analysis (Tables S7 and S8). Therefore, to further assess the robustness of the six‐snoRNA signature, we performed data stratification analysis to estimate whether the six‐snoRNA signature exhibited prognostic value within the same clinical factor.

**Figure 3:**
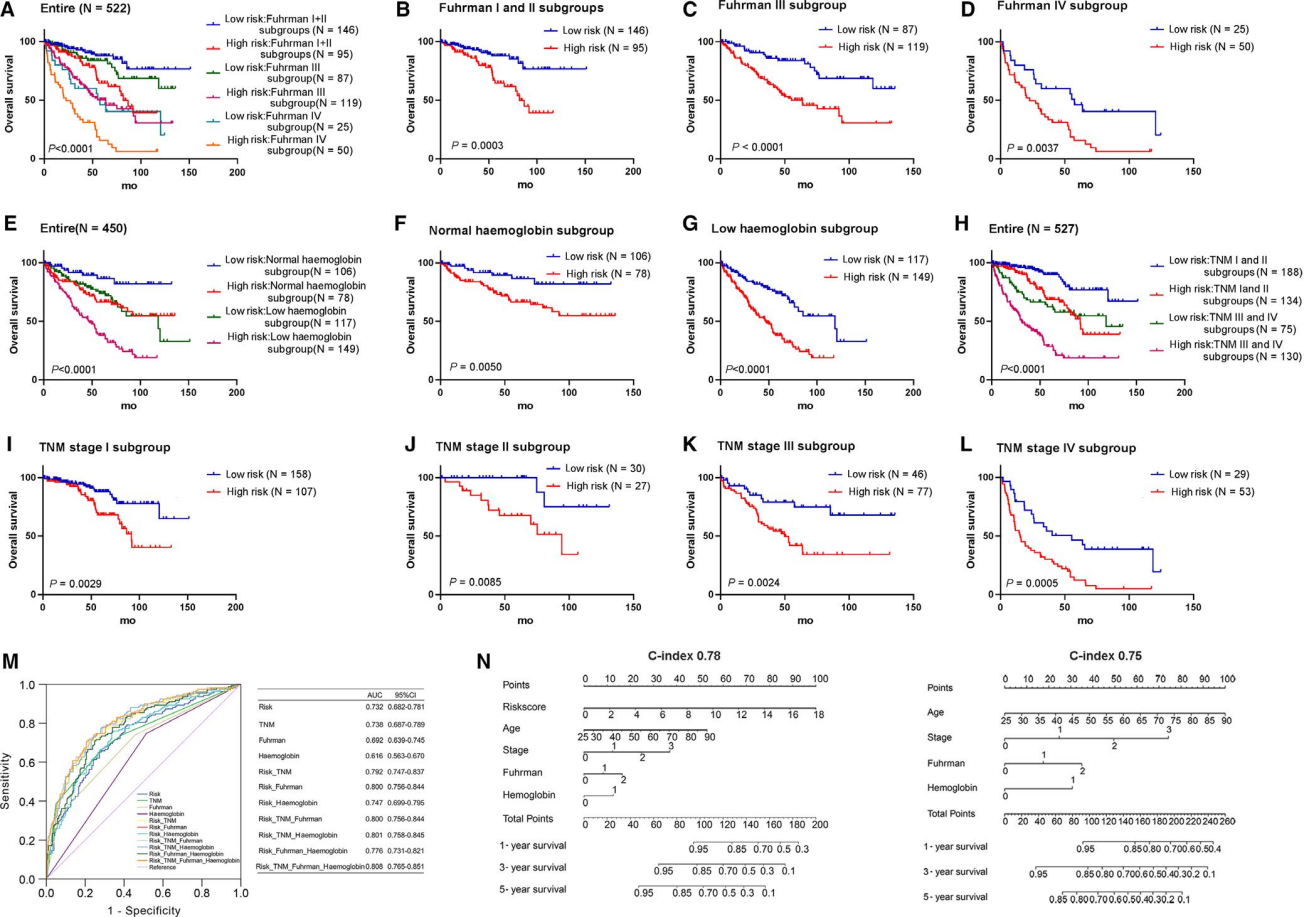
Kaplan‐Meier estimates of the overall survival of TCGA patients using the six‐snoRNA signature. A, Kaplan‐Meier curves for the entire TCGA series patients with Fuhrman (I and II, III, and IV). Kaplan‐Meier curves for patients with Fuhrman I and II (B), Fuhrman III (C) and Fuhrman IV (D). E, Kaplan‐Meier curves for the entire TCGA series patients with haemoglobin (combined with normal and low‐level haemoglobin). Kaplan‐Meier curves for patients with normal haemoglobin (F) and low‐level haemoglobin (G). H, Kaplan‐Meier curves for the entire TCGA series patients with TNM stage (I and II, III and IV). Kaplan‐Meier curves for patients with TNM stage I (I), TNM stage II (J), TNM stage III (K) and TNM stage IV (L). M, ROC analysis of the sensitivity and specificity of the overall survival prediction by the six‐snoRNA risk score, TNM stage, Fuhrman and haemoglobin. N, The nomogram for predicting proportion of patients with 1‐, 3‐ and 5‐y OS (stage: 0 = stage I, 1 = stage II, 2 = stage III and 3 = stage IV; Fuhrman grade: 0 = Fuhrman grade I + II, 1 = Fuhrman grade III and 2 = Fuhrman grade IV; and haemoglobin: 0 = normal haemoglobin level and 1 = low haemoglobin level)

The Fuhrman grading system was an important independent and most widely used predictive factor in renal cell carcinoma, based on assessment of the uniformity of nuclear size, nuclear shape and nuclear prominence.^26^, ^27^, ^28^ Hence, we first stratified patients into Fuhrman I and II groups, III group and IV group. Then, we divided these patients into high‐risk and low‐risk groups again. As results shown in Figure 3A, patients in high‐risk group had significantly shorter OS than those in low‐risk group no matter in Fuhrman I and II groups or in Fuhrman III group and IV group (*P* < .0001). Furthermore, we separately analysed Fuhrman I and II groups, III group and IV group, and found after the six‐snoRNA signature separated them into high‐risk and low‐risk groups, patients in high‐risk group had shorter OS (Figure 3B‐D). In addition, patients in high‐risk group had shorter RFS than those in low‐risk group which was the same with OS, although there was no statistically significant in Fuhrman IV subgroup (Figure S1C).

For ccRCC, according to the Memorial Sloan‐Kettering Cancer Center (MSKCC) criteria, serum haemoglobin less than the lower limit of normal (LLN) was one of the adverse prognostic factors.^29^, ^30^ Hence, we first stratified patients into normal and low‐level haemoglobin groups. Then, we divided these patients into high‐risk and low‐risk groups again. As results shown in Figure 3E, patients in high‐risk group had significantly shorter OS than those in low‐risk group no matter in normal or in low‐level haemoglobin group (*P* < .0001). Furthermore, we separately analysed normal and low‐level haemoglobin groups, and found after the six‐snoRNA signature separated them into high‐risk and low‐risk groups, patients in high‐risk group had shorter OS (Figure 3F‐G). In addition, patients in high‐risk group had shorter RFS than those in low‐risk group which was the same with OS (Figure S1C).

Lastly, we first stratified patients into TNM I and II stage groups and TNM III and IV stage groups according to TNM stage. Then, we divided these patients into high‐risk and low‐risk groups again. As results shown in Figure 3H, patients in high‐risk group had significantly shorter OS than those in low‐risk group no matter in TNM I and II stages or TNM III and IV stages (*P* < .0001). Furthermore, we separately analysed I stage, II stage, III stage and IV stage, and found after the six‐snoRNA signature clearly separated them into high‐risk and low‐risk groups, patients in high‐risk group had shorter OS (Figure 3I‐L). Besides patients in high‐risk group had shorter RFS than those in low‐risk group which was the same with OS, there were no statistically significant in TNM stage II subgroup and TNM stage III subgroup (Figure S1C).

Combining the results of multivariable Cox regression analysis and stratification analysis, the six‐snoRNA signature was demonstrated to be a powerful and independent indicator for survival prediction in ccRCC patients.

To compare the sensitivity and specificity of predictive ability among the six‐snoRNA signature, TNM stage, Fuhrman grade and haemoglobin level, we performed receiver operating characteristic curve analysis (ROC). The area under characteristic (AUC) was calculated and compared among the four independent prognostic factors (Figure 3M). Firstly, as an independent indicator of survival prediction for ccRCC patients, the six‐snoRNA signature (AUC = 0.732, *P* < .0001) showed higher AUC than Fuhrman grade (AUC = 0.692, *P* < .0001) and haemoglobin level (AUC = 0.616, *P* < .0001). Moreover, the sensitivity and specificity of the six‐snoRNA signature (AUC = 0.732, *P* < .0001) were as good as TNM stage (AUC = 0.738, *P* < .0001). Previous studies have reported that combined biomarkers were able to improve the prognostic accuracy than a single biomarker.^31^ Therefore, we further determined whether the incorporation of risk score into clinical indicators could better predict prognosis of ccRCC patients. The result showed that when the six‐snoRNA signature incorporated into TNM stage, Fuhrman grade or haemoglobin, the AUC of three clinical characteristics increased from 0.738, 0.692 and 0.616 to 0.792, 0.800 and 0.747, respectively. Although we had calculated AUC when three or four independent risk factors combined together, there was no obvious optimization compared with the six‐snoRNA signature and TNM stage or the six‐snoRNA signature and Fuhrman grade combination. These results suggested the six‐snoRNA signature combining with TNM stage or Fuhrman grade probably processed the clinical applied value for ccRCC.

Based on univariate and multivariate Cox regression analysis, we further performed nomogram analysis of independent prognosis factors and compared the C‐index value with or without our risk score system. We found when risk score, age, TNM stage, Fuhrman grade and haemoglobin were included, the C‐index of nomogram was 0.78 (95% CI = 0.74‐0.82) and when age, TNM stage, Fuhrman grade and haemoglobin were included, the C‐index was 0.75 (95% CI = 0.71‐0.78) (Figure 3N). This result demonstrated our risk score system was effective and would be a good index parameter in prognostic prediction.

### 3.4 Validating the prognostic value of the six‐snoRNA signature in ccRCC tissue and serum

**Figure 4:**
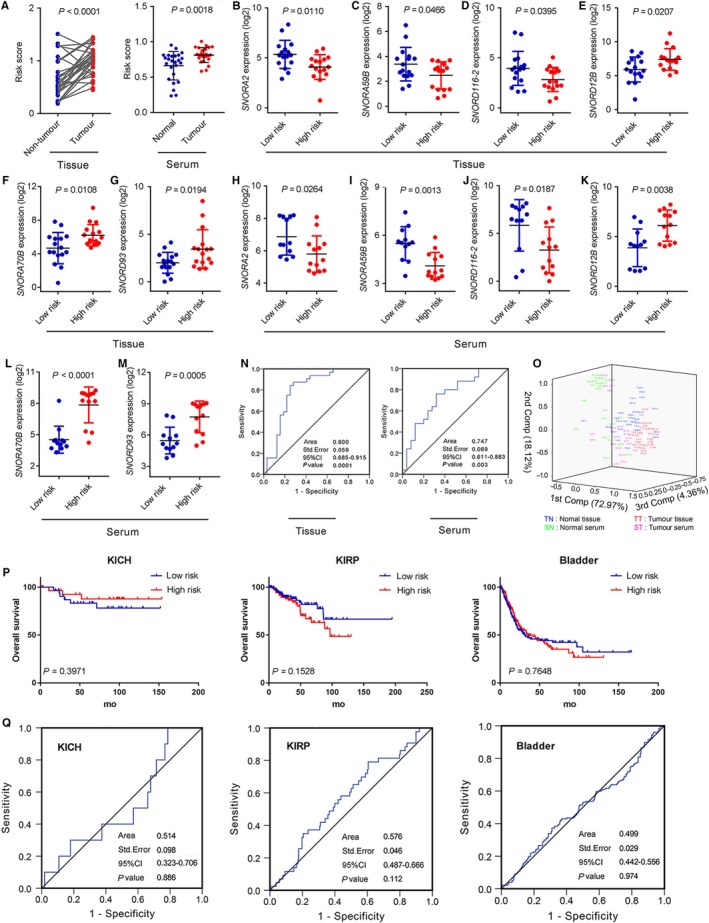
The expressions of six snoRNAs and risk score in ccRCC patients’ tissues and serums. A, The comparisons of six‐snoRNA risk score for ccRCC tissues and serums, respectively. The expressions of six snoRNAs in tissues (B‐G) and serums (H‐M). *P* values were measured by unpaired *t* test. N, ROC analysis of the sensitivity and specificity of the overall survival prediction by the six‐snoRNA risk score in tissues and serums. O, 3‐D PCA plot analysis of the six‐snoRNA expression data in tissues and serums samples. Abbreviations; TN: normal tissue, TT: tumour tissue, SN: normal serum, ST: tumour serum. P, Kaplan‐Meier curves for TCGA patients with KICH, KIRP and bladder cancer by the six‐snoRNA risk score. Q, ROC analysis of the sensitivity and specificity of the overall survival prediction for patients with KICH, KIRP and bladder cancer by the six‐snoRNA risk score

To further validate the prognostic value of the six‐snoRNA signature for ccRCC, we, respectively, measured these six‐snoRNA expressions in ccRCC patients and healthy people by qRT‐PCR. Then, we calculated and compared the risk score of patients in tissue and serum, respectively, and found that the risk score was significantly higher in patients' tissue and serum, compared with normal controls (*P* < .0001, *P* = .0018, Figure 4A). Furthermore, no matter in tissue or in serum samples, patients in high‐risk group exhibited higher expression of risky snoRNAs, whereas patients in low‐risk group exhibited higher expression of protective snoRNAs (Figure 4B‐M). Taken together, these results suggested that the six‐snoRNA signature exhibited its diagnostic value as biomarker for ccRCC patients.

Furthermore, to evaluate the sensitivity and specificity of the six‐snoRNA signature in distinguishing patients from healthy individuals, ROC analysis was performed and the AUC was calculated using tissue and serum expression of each snoRNA. The results showed that the AUC value was 0.800 in tissue and 0.747 in serum (Figure 4N), suggesting the six‐snoRNA signature had high sensitivity and specificity for ccRCC in tissue and serum. To further compare the diagnostic performance among the six‐snoRNA signature between tissue and serum, and evaluate the overall quality of our samples, the 3‐D PCA plot (Figure 4O) was performed and showed that the tissue samples and serum samples could be separated by the 1st component, which explained 72.97% of the data variation. Furthermore, the normal tissue and tumour tissue scattered separately on the 1st component, and the normal serum and tumour serum were scattered separately by the 2nd component, which explained 18.12% of the data variation. Also, the 3‐D PCA plot showed that the reproducibility of tissue samples was better than serum samples. In addition, we further investigated whether there was a relationship between the risk score and clinicopathologic characteristics in tissue and found that higher risk score was significantly associated with advanced TNM stage and higher Fuhrman grade (Table S9). Taken together, these results demonstrated that the six‐snoRNA signature yielded better diagnostic accuracy in distinguishing patients from normal controls.

Although the six‐snoRNA signature served as a potentially useful indicator for assessing poor prognosis of ccRCC patients, it was unclear whether the six‐snoRNA signature was working in other urological malignancies. Therefore, we analysed expression data sets and corresponding clinical and survival data of other urological malignancies including KICH, KIRP and bladder cancer in TCGA database. As results shown in Figure 4P, there was no difference in OS in high‐risk group and low‐risk group for KICH (*P* = .3971), KIRP (*P* = .1582) and bladder cancer (*P* = .7648). Simultaneously, we also performed ROC analysis and found that the six‐snoRNA signature had low sensitivity and specificity of survival prediction for KICH (AUC = 0.514), KIRP (AUC = 0.576) and bladder cancer (AUC = 0.499) (Figure 4Q), indicating the six‐snoRNA signature could not be used as biomarker for these cancers. These results demonstrated that the six‐snoRNA signature served as an effective and specific biomarker to predict the survival of ccRCC.

### 3.5 The snoRNA methylation and WNT pathway potentially function ccRCC tumorigenesis

**Figure 5:**
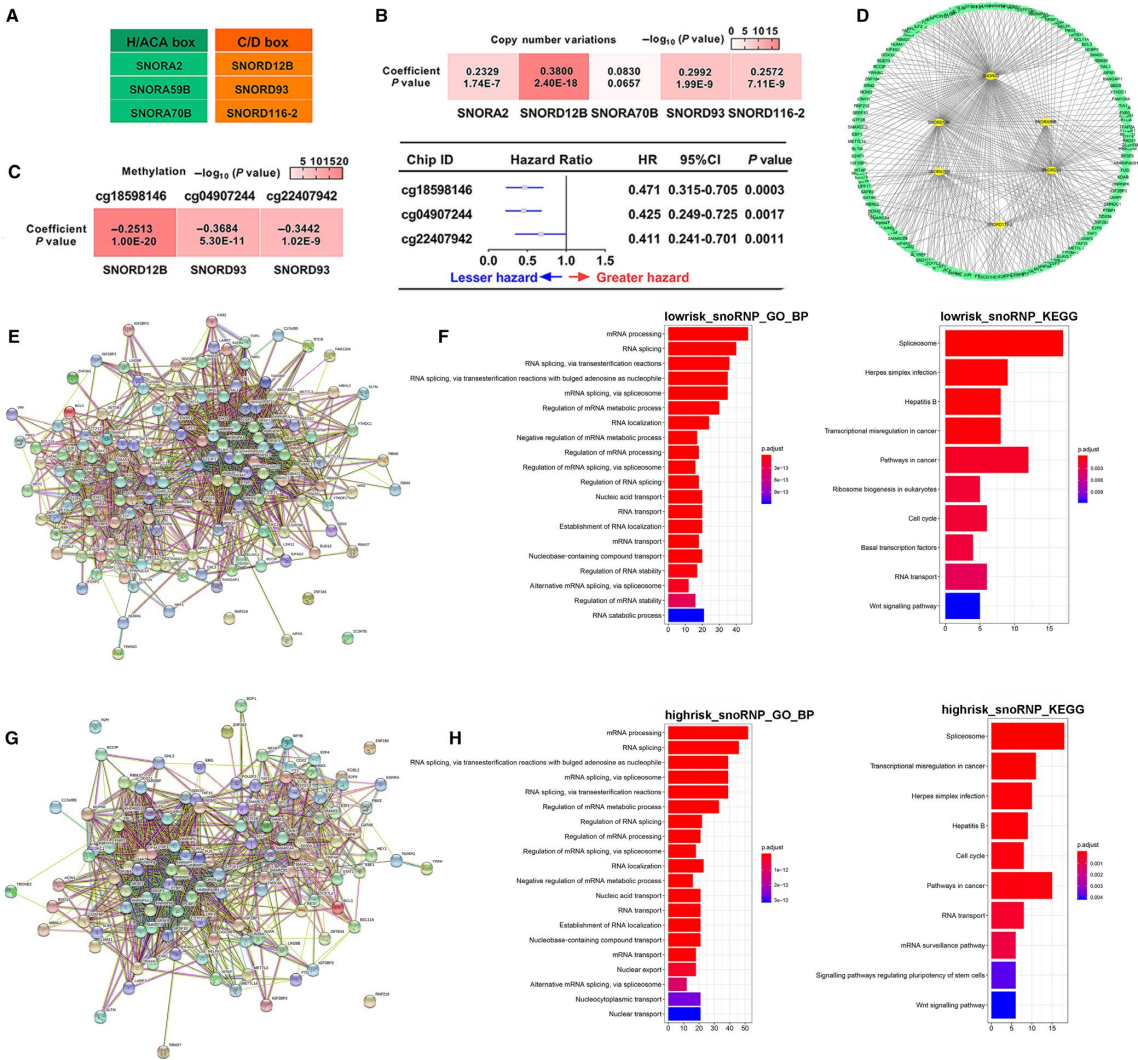
The function analysis of these six snoRNAs. A, Distribution of different types of snoRNAs. B, The correlation between snoRNAs and copy number variations. C, The correlation between snoRNAs and methylation and multivariable cox analysis of methylation site in ccRCC. D, Network of snoRNP genes highly associated with six snoRNAs. Protein‐protein interactions analysis of snoRNP genes highly correlated with low‐risk snoRNAs (E) and high‐risk snoRNAs (G) by STRING tool. Biological process analysis and KEGG analysis of snoRNP genes highly correlated with low‐risk snoRNAs (F) and high‐risk snoRNAs (H) by clusterProfiler package

The biological function of snoRNAs has not been investigated clearly. Small nucleolar RNAs are primarily classified into H/ACA box and C/D box snoRNAs based on their structure and main function. H/ACA box snoRNAs guide pseudouridylation of nucleotides, whereas C/D box snoRNAs are responsible for 2′‐O‐methylation.^11^, ^12^, ^13^ As shown in Figure 5A, among these selected six snoRNAs, there were three C/D box snoRNAs including *SNORD12B*, *SNORD93* and *SNORD116‐2*, and three were H/ACA box snoRNAs including *SNORA2*, *SNORA59B* and *SNORA70B*. Copy number variation (CNV) is a key regulator of gene expression, and some snoRNAs were significantly associated with their CNVs in various cancers.^19^, ^32^, ^33^ Therefore, we evaluated relevance of CNV and these snoRNA expressions. The result showed that the expression levels of *SNORA2*, *SNORD12B*, *SNORA70B*, *SNORD93* and *SNORD116‐2* were positively correlated with their CNVs in ccRCC, respectively (Figure 5B). In addition, DNA methylation is a common epigenetic mechanism that regulates gene expression and studies have reported that the methylation level of snoRNAs was involved in regulating snoRNA expression.^19^, ^34^, ^35^ Thus, we further evaluated the correlation of these snoRNAs with DNA methylation using snoRic database. The results showed *SNORD12B* and *SNORD93* had significant correlation with their DNA methylation (Figure 5C). To illustrate, we have validated *SNORD12B* was the risk factor in ccRCC, and the expression of *SNORD12B* was negatively correlated with the methylation level of probe cg18598146. Hence interestingly, probe cg18598146 methylation of *SNORD12B* was protective factor for ccRCC (Figure 5C). Similar result was also observed in *SNORD93*. These results demonstrated that the methylation level of snoRNAs might affect prognosis of ccRCC by regulating the expression level of snoRNAs.

In eukaryotes, the complexes between snoRNAs and ribonucleoproteins (RNPs) are called small nucleolar RNPs (snoRNPs).^36^ Previous report suggested that RNPs were strongly correlated with snoRNAs and demonstrated their co‐activation and synergy involves in cancer progression by affecting the processes of ribosome and protein translation.^19^ Therefore, we further investigated the correlations between these six snoRNAs and their corresponding RNPs. We identified 159 RNPs that were associated with these six snoRNAs (Figure 5D). To further explore the potential function of these snoRNAs, we first performed protein‐protein interaction analysis for these snoRNPs by STRING tool (Figure 5E,G). Then, we performed GO and KEGG enrichment analysis by clusterProfiler package to analyse high‐risk group‐related snoRNP genes genes and low‐risk group related‐snoRNP genes, respectively. We found most high‐risk group‐related snoRNP genes genes were similar to low‐risk group‐related snoRNP genes (Table S10). The GO analysis results showed that high‐risk–related snoRNPs (Figure 5H) and low‐risk–related snoRNPs (Figure 5F) were mainly enriched in ‘mRNA processing’, ‘RNA splicing’ and so on. The KEGG analysis results showed that high‐risk–related snoRNPs (Figure 5H) and low‐risk–related snoRNPs (Figure 5F) were mainly enriched in ‘Splicesome’, ‘Transcription misregulation in cancer’, ‘cell cycle’, ‘Wnt signalling pathway’ and so on. These results suggested that there was no significant difference between high‐risk–related snoRNPs and low‐risk–related snoRNPs.

**Figure 6:**
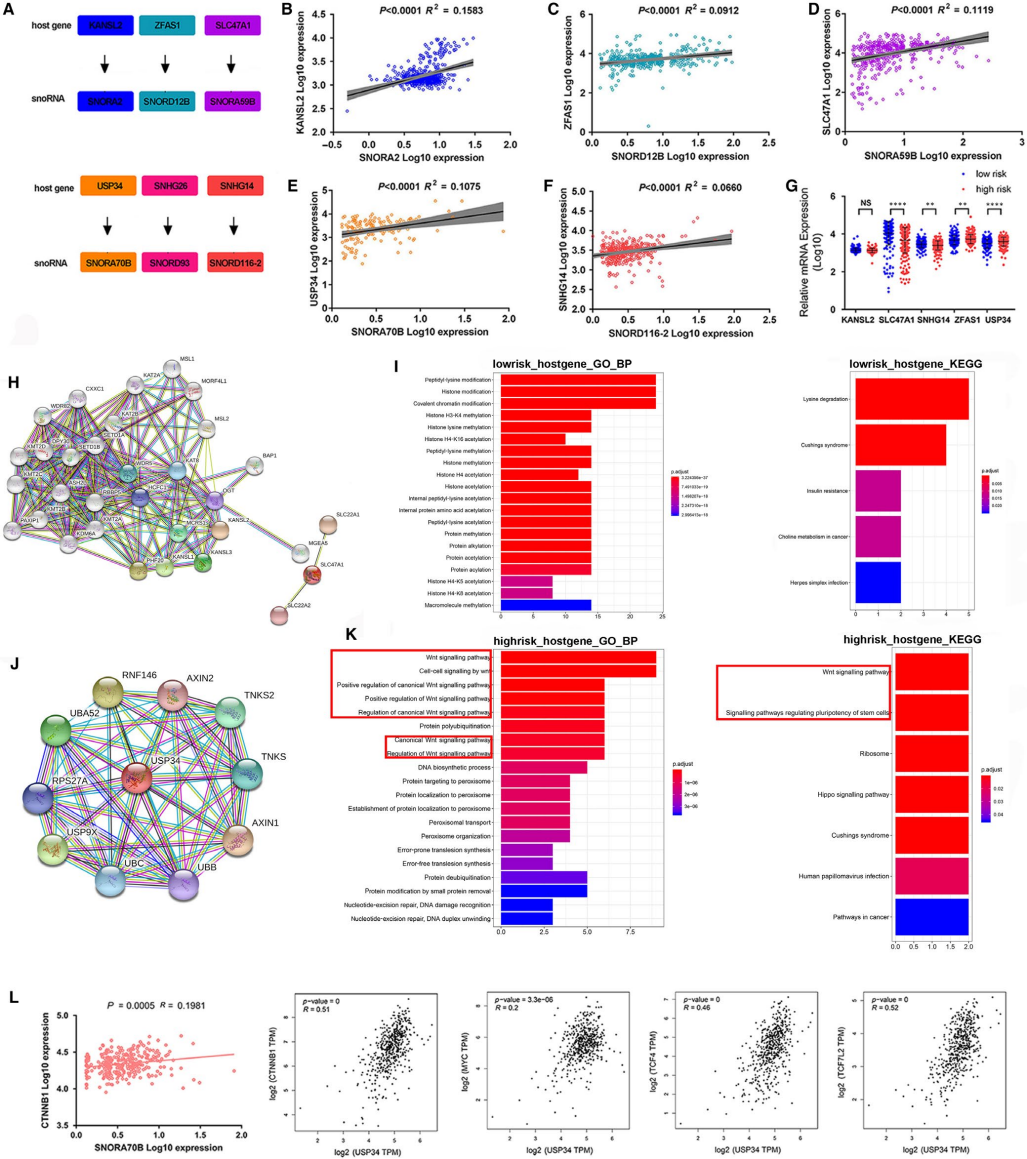
The function analysis of the host genes of these six snoRNAs. A, The host genes of six snoRNAs. (B‐F) The correlation between host genes and snoRNAs. G, The differential expression of snoRNAs' host genes between high‐risk and low‐risk groups. Protein‐protein interaction analysis of snoRNP genes highly correlated with low‐risk snoRNAs (H) and high‐risk snoRNAs (J) by STRING tool. Biological process analysis and KEGG analysis of snoRNP genes highly correlated with low‐risk snoRNAs (I) and high‐risk snoRNAs (K) by clusterProfiler package. (L) The correlation between *SNORA70B* and *CTNBB1*, and between *SNORA70B* host gene *USP34* and *CTNBB1*, *MYC*, *TCF4* and *TCF7L2*

In mammals, the majority of snoRNAs are encoded within introns of protein‐coding or non‐coding genes, which are called ‘host genes’.^37^ An alteration of snoRNA expression may result from host genes through co‐transcription.^38^ To understand the potential regulated mechanism of snoRNA expression, we further analysed the distribution and function of host genes for these snoRNAs. We observed that host genes of *SNORA2*, *SNORA59B* and *SNORA70B* were protein‐coding genes and host genes of *SNORD12B*, *SNORD93* and *SNORD116‐2* were non‐coding genes (Figure 6A). Then, we analysed the correlation between snoRNAs and their corresponding host genes, and observed that *SNORA2*, *SNORD12B*, *SNORA59B*, *SNORA70B* and *SNORD116‐2* were highly correlated with their host genes, respectively (Figure 6B‐F). In addition, we also analysed differential expression of host genes between low‐risk and high‐risk groups (Figure 6G). The results showed that *SLC47A1* and *SNHG14*, host genes of protective *SNORA59B* and *SNORD116‐2*, had higher expression in low‐risk group and *ZFAS1* and *USP34*, host genes of risky *SNORD12B* and *SNORA70B,* had higher expression in high‐risk group. These results suggested that the host genes of snoRNA may play the same protective or risky role as well as snoRNA itself. To explore the potential function of these protein‐coding host genes, we first performed protein‐protein interactions analysis for these host genes by STRING tool (Figure 6H,J). Then, we performed GO and KEGG enrichment analysis by clusterProfiler package to analyse high‐risk group–related host genes and low‐risk group–related host genes, respectively. The GO analysis results showed that low‐risk–related host genes were mainly enriched in ‘peptidyl‐lysine modification’, ‘histone modification’ and so on (Figure 6I) and high‐risk–related host genes were mainly enriched in ‘Wnt signalling pathway’, ‘cell‐cell signalling by wnt’ and so on (Figure 6K). The KEGG analysis results showed that low‐risk–related host genes were mainly enriched in ‘Lysine degradation’, ‘Cushing's syndrome’ and so on (Figure 6I), and high‐risk–related host genes were mainly enriched in ‘Ribosome’, ‘Wnt signalling pathway’ and so on (Figure 6K). Both GO and KEGG analysis results showed that high‐risk–related host genes were mainly enriched in ‘Wnt signalling pathway’. Hence, we further analysed correlation between *SNORA70B*, its host gene *USP34* and Wnt signalling pathway–related genes such as *CTNNB1*, *MYC*, *TCF4* and *TCF7L2* (Figure 6L). The results showed that *SNORA70B* was positively related to *CTNNB1* and *USP34* was positively related to *CTNNB1*, *MYC*, *TCF4* and *TCF7L2*, suggesting *SNORA70B* and its host gene *USP34* might play significant roles in ccRCC tumorigenesis through ‘Wnt signalling pathway’.

## 4 DISCUSSION

Clear cell renal cell carcinoma is the most common subtype among renal cell carcinoma.^2^, ^39^ In the past few decades, great progress has been made from a non‐specific immune approach to targeted therapy (against VEGF, PDGF) and now to novel immunotherapy with immune‐checkpoint inhibitors.^31^ At present, indolent and aggressive tumours cannot be distinguished depending on TNM staging system, which mainly relies on anatomical information without biological characteristics. Although *APEX1* has been reported diagnosis value of ccRCC, its clinical practical prospective was still a long way.^40^ In recent years, the potential of snoRNA as biomarkers has been graduated recognized.^15^, ^16^, ^41^ For example, *SNORA42* was identified as a novel diagnostic, predictive biomarker and prospective therapeutic targets for CRC patients.^15^ In our study, we identified a six‐snoRNA signature as the diagnosis marker depending on TCGA database and validated its value to distinguish ccRCC patients and healthy people in tissue and serum samples.

TNM stage, Fuhrman grade and haemoglobin level are conventional clinical‐pathological characteristics for ccRCC. Although these features are very important, they are not just belong to ccRCC. There is still a desert of specific diagnostic indicators in ccRCC Gong et al have reported snoRNAs were specifically overexpression in ccRCC, implying the potential value of snoRNAs as biomarkers. In our study, we found the six‐snoRNA signature was an independent risk factor for OS and RFS in ccRCC (Tables S6 and S8), and high six‐snoRNA signature expression indicated poor OS and RFS. In addition, we also compared the performance between conventional clinical‐pathological characteristics and our six‐snoRNA signature. Intriguingly, we found six‐snoRNA signature was as good as TNM stage and much better than Fuhrman grade and haemoglobin level. Besides, we further integrated the six‐snoRNA signature with TNM stage or Fuhrman grade and found their potentially clinical applied value. More significantly, we confirmed the six‐snoRNA signature was related to clinical characteristics in tissue, especially TNM stage and Fuhrman grade.

Minimal invasion, easy method for detection, low cost and convenient census are the advantages of serum‐based biomarkers. The previous study has reported snoRNAs were stably present and reliably detectable in serum and suggested snoRNAs could be as novel non‐invasive diagnostic biomarkers for osteoarthritis.^42^ However, the expression of snoRNA in ccRCC serum has not been studied. Hence, we further investigated the six‐snoRNA signature's stability in serum to evaluate the clinical potential value in ccRCC. In our study, we found the six‐snoRNA signature expressed stably in serum, suggesting serum snoRNAs may serve as novel non‐invasive biomarkers for ccRCC. The results were similar to above TCGA data set analysis, verifying the feasibility and validity of the six‐snoRNA signature as molecular marker. Interestingly, according to the results of ROC and 3‐D PCA, we found the six‐snoRNA signature performance in tissue was better than that in serum. The possible reasons for these results may be as follows: (a) Although snoRNA itself was stable, it was not that high level in serum. Therefore, our detection methods needed to be further optimized; (b) at present, the method of extracting snoRNA in serum was not perfect enough, resulting in more snoRNA loss during the extraction process; and (c) the number of serum samples was much smaller than tissue samples. It should be pointed out that although expression profile‐based snoRNA signature with superiority for survival prediction in ccRCC patients has not yet been applied in clinical practice, this area seems prospective with the development of technology.

An increasing number of studies emerged about the mechanism research of snoRNA regulating the development of cancer. Hypermethylation is characteristic of most ccRCCs.^43^ On the one hand, the C/D box snoRNAs exert a promotion role in tumorigenesis by regulating rRNA 2′‐O‐methylation.^44^, ^45^ *SNORD12B* and *SNORD93* in our selected snoRNAs were C/D box snoRNAs and exhibited a tumorigenic effect in ccRCC. On the other hand, snoRNAs exist methylation site and have poor prognosis in KIRC, so is snoRNA methylation correlated with better survival?^19^ In this study, we found high expression of risky factors *SNORD12B* and *SNORD93* was negatively correlated with methylation sites cg18598146, cg04907244 and cg22407942, respectively, and these methylation sites were associated with better survival in ccRCC. In addition, Ferreira et al^46^ suggested that the host gene–associated 5'CpG islands of *SNORA59B* were hypermethylated in colorectal cancer cells. Hence, the specific function of methylation in snoRNAs regulating ccRCC is paradox and further investigation is needed.

WNT family genes play important roles in human organogenesis and tumorigenesis. Moreover, they were involved in renal development and initiation of several renal diseases including kidney malignancy.^47^ In our study, host gene *USP34* of *SNORA70B* was mainly involved in regulating WNT signalling pathway. Interestingly, we did not found *SNORA70B*‐related snoRNPs, which was the most studied mechanism, participated in WNT pathway. Lasted studies have reported that snoRNAs could directly bind to functional protein to promote tumorigenesis.^48^, ^49^ Therefore, we inferred that *USP34* or *SNORA70B* might bind WNT pathway–related protein to activate it.

In general, we identified a six‐snoRNA signature as an independent and specific indicator to diagnose and predict prognosis of ccRCC patients, providing a prospective diagnostic and prognostic biomarker and potential therapeutic targets for ccRCC.

## CONFLICTS OF INTEREST

The authors declare no potential conflicts of interest.

## **AUTHORS**'** CONTRIBUTIONS**

YZ and YY contribute equally to the study. YY, YZ, MW and MH designed the study and wrote the paper. YZ and RM performed qRT‐PCR. MW, MH, BY, LW and JW provided samples. YC, RM, XL, LZ, WZ, LZ, LJ and LZ analysed data. All authors have read and approved the final version of the manuscript.

## ETHICAL APPROVAL

All patients consented to an institutional review board–approved protocol that allows comprehensive analysis of tumour samples (Ethics committee of the Second and Fourth Affiliated Hospitals of China Medical University and the Second Hospital of Dalian Medical University).

## Data Availability

The data sets used and analysed during the current study are available from the corresponding author on reasonable request.
