# Supplementary figures and images for: Expression signature of six‐snoRNA serves as novel non‐invasive biomarker for diagnosis and prognosis prediction of renal clear cell carcinoma
Source: J Cell Mol Med. 2020 Jan 14;24(3):2215–28. doi: 10.1111/jcmm.14886 (PMC7011154; doi:10.1111/jcmm.14886)

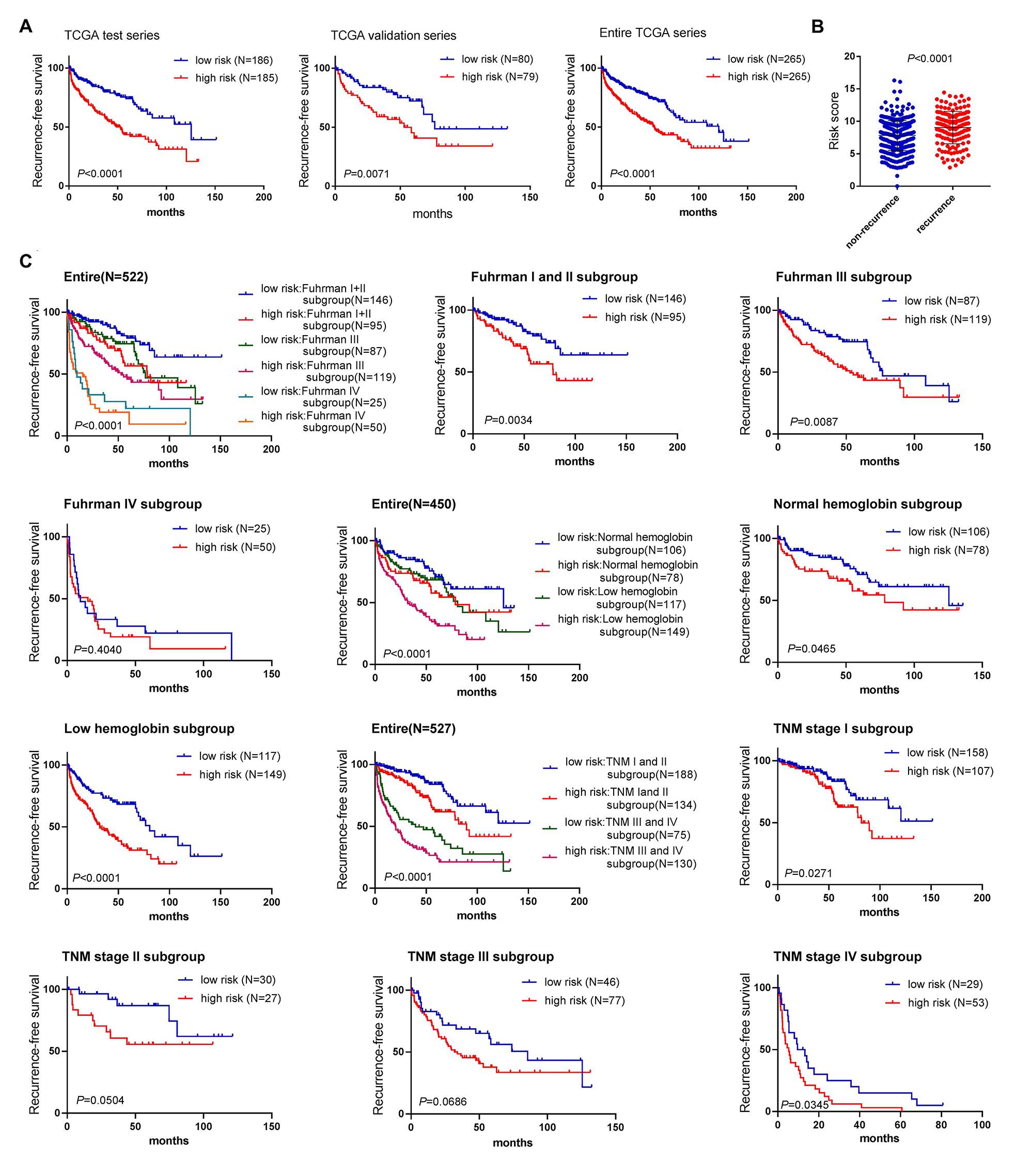

Supplement: Supplementary file 1 [file JCMM-24-2215-s001.tif]
