# Supplementary material for: Expression signature of six‐snoRNA serves as novel non‐invasive biomarker for diagnosis and prognosis prediction of renal clear cell carcinoma
Source: J Cell Mol Med. 2020 Jan 14;24(3):2215–28. doi: 10.1111/jcmm.14886 (PMC7011154; doi:10.1111/jcmm.14886)
Supplement: Supplementary file 2 [file JCMM-24-2215-s002.docx]

**Table S1. Clinical characteristics of ccRCC in each data set**

| **Characteristics** | **Test series** | **Validation series** | **Entire series** |
| --- | --- | --- | --- |
| Gender |  |  |  |
| Male | 239/371 (64.4%) | 105/159 (66.0%) | 344/530 (64.9%) |
| Female | 132/371 (35.6%) | 54/159 (34.0%) | 186/530 (35.1%) |
| Age (years) |  |  |  |
| >65 | 140/371 (37.7%) | 117/159 (73.6%) | 257/530 (48.5%) |
| ≤65 | 231/371 (62.3%) | 42/159 (26.4%) | 273/530 (51.5%) |
| TNM stage |  |  |  |
| I | 187/369 (50.7%) | 78/158 (49.4%) | 265/527 (50.3%) |
| II | 36/369 (9.8%) | 21/158 (13.3%) | 57/527 (10.8%) |
| III | 93/369 (25.2%) | 30/158 (19.0%) | 123/527 (23.3%) |
| IV | 53/369 (14.4%) | 29/158 (18.4%) | 82/527 (15.6%) |
| Fuhrman grade |  |  |  |
| I | 11/366 (3.0%) | 3/156 (1.9%) | 14/522 (2.7%) |
| II | 160/366 (43.7%) | 67/156 (42.9%) | 227/522 (43.5%) |
| III | 142/366 (38.8%) | 64/156 (41.0%) | 206/522 (39.5%) |
| IV | 53/366 (14.5%) | 22/156 (14.1%) | 75/522 (14.4%) |
| Hemoglobin level |  |  |  |
| Normal level | 127/317 (40.1%) | 57/133 (42.9%) | 184/450 (40.9%) |
| Low level | 190/317 (59.9%) | 76/133 (57.1%) | 266/450 (59.1%) |
