# Supplementary material for: Expression signature of six‐snoRNA serves as novel non‐invasive biomarker for diagnosis and prognosis prediction of renal clear cell carcinoma
Source: J Cell Mol Med. 2020 Jan 14;24(3):2215–28. doi: 10.1111/jcmm.14886 (PMC7011154; doi:10.1111/jcmm.14886)
Supplement: Supplementary file 3 [file JCMM-24-2215-s003.docx]

**Table S2. The detailed information of differential snoRNAs**

| Gene symbol | Log FC | *P* value | Gene symbol | Log FC | *P* value |
| --- | --- | --- | --- | --- | --- |
| SNORA70F | -2.5482 | 6.06E-78 | SNORD117 | 1.0942 | 8.19E-08 |
| SNORA2 | -1.4426 | 1.24E-35 | SNORA53 | 1.6143 | 8.96E-08 |
| SNORD116-24 | -1.7053 | 3.26E-30 | SNORA59B | 1.3228 | 1.16E-07 |
| SNORD116-4 | -1.4445 | 8.47E-21 | snoZ196 | 1.1115 | 1.85E-07 |
| SNORD116-2 | -1.4843 | 3.75E-13 | SNORD17 | 1.3405 | 7.08E-07 |
| SNORD116-26 | -1.4201 | 2.01E-10 | SNORD15B | 1.2937 | 1.39E-06 |
| SNORD116-1 | -1.2854 | 4.83E-10 | SNORA14 | 1.4728 | 2.79E-05 |
| SNORD116-27 | -1.1177 | 3.54E-07 | SNORD12C | 1.0284 | 4.93E-05 |
| SNORA80B | -1.0109 | 3.34E-06 | SNORA70B | 1.2244 | 7.07E-05 |
| SNORD99 | 2.2038 | 1.25E-23 | SNORD88A | 1.3385 | 7.70E-05 |
| SNORD60 | 1.6403 | 4.50E-15 | SNORA7B | 1.1075 | 8.88E-05 |
| SNORD104 | 1.3677 | 3.82E-14 | SNORA75 | 1.1226 | 0.000177 |
| SNORA73B | 2.3260 | 1.65E-12 | SNORA54 | 1.3882 | 0.000389 |
| SNORD123 | 1.6319 | 2.45E-12 | SNORA74A | 1.7648 | 0.000528 |
| SNORD63 | 1.0274 | 3.31E-12 | SNORA74 | 1.3414 | 0.000653 |
| SNORA16 | 1.2376 | 4.60E-11 | SNORA23 | 1.1356 | 0.000947 |
| SNORA71A | 1.1964 | 9.11E-11 | SNORA84 | 1.5594 | 0.001096 |
| SNORD93 | 1.5416 | 3.85E-10 | SNORA70G | 1.1147 | 0.001359 |
| SNORA71C | 1.0838 | 6.35E-10 | SNORA38 | 1.2584 | 0.003894 |
| SNORA7 | 1.1402 | 9.39E-09 | SNORA71D | 1.2961 | 0.004624 |
| SNORD124 | 1.2044 | 2.19E-08 | SNORA49 | 1.3034 | 0.004657 |
| SNORD12B | 1.0126 | 2.41E-08 |  |  |  |
