# Supplementary material for: Expression signature of six‐snoRNA serves as novel non‐invasive biomarker for diagnosis and prognosis prediction of renal clear cell carcinoma
Source: J Cell Mol Med. 2020 Jan 14;24(3):2215–28. doi: 10.1111/jcmm.14886 (PMC7011154; doi:10.1111/jcmm.14886)
Supplement: Supplementary file 4 [file JCMM-24-2215-s004.docx]

**Table S3. Univariable Cox regression analysis to access the prognostic value of each snoRNA**

| **Gene symbol** | **HR^a^** | **95%CI** | ***P*value^b^** |
| --- | --- | --- | --- |
| *SNORA70B* | 1.6108 | 1.4437-1.7779 | 2.26E-08 |
| *SNORA7* | 1.3690 | 1.0630-1.6749 | 0.0442 |
| *SNORA14* | 1.2841 | 1.1073-1.4610 | 0.0056 |
| *snoZ196* | 1.2840 | 1.1009-1.4673 | 0.0075 |
| *SNORA73B* | 1.2449 | 1.1265-1.3633 | 0.0003 |
| *SNORD93* | 1.2444 | 1.0477-1.4411 | 0.0294 |
| *SNORA71A* | 1.2343 | 1.0537-1.4148 | 0.0224 |
| *SNORD99* | 1.2297 | 1.0996-1.3599 | 0.0018 |
| *SNORD12B* | 1.2163 | 1.0341-1.3984 | 0.0352 |
| *SNORA74A* | 1.2087 | 1.0342-1.3831 | 0.0333 |
| *SNORA59B* | 0.8217 | 0.6915-0.9518 | 0.0031 |
| *SNORD116-24* | 0.7870 | 0.6508-0.9232 | 0.0006 |
| *SNORD116-4* | 0.7524 | 0.6157-0.8891 | 4.52E-05 |
| *SNORD116-27* | 0.7199 | 0.4400-0.9997 | 0.0213 |
| *SNORA2* | 0.6665 | 0.4031-0.8628 | 3.34E-05 |
| *SNORD116-2* | 0.6329 | 0.4031-0.8628 | 9.64E-05 |

Abbreviation: HR, hazard ratio.

^a^Values >1.0 indicate that expression is positively associated with poor survival.

^b^Likelihood ratio test *P* value.
