# Supplementary material for: Expression signature of six‐snoRNA serves as novel non‐invasive biomarker for diagnosis and prognosis prediction of renal clear cell carcinoma
Source: J Cell Mol Med. 2020 Jan 14;24(3):2215–28. doi: 10.1111/jcmm.14886 (PMC7011154; doi:10.1111/jcmm.14886)
Supplement: Supplementary file 5 [file JCMM-24-2215-s005.docx]

**Table S4. The HR and *P* value of each snoRNA**

| **Gene symbol** | **Coefficient**^a^ | **HR**^b^ | **95%CI** | ***P* value**^c^ |
| --- | --- | --- | --- | --- |
| *SNORA70B* | 0.4199 | 1.5219 | 1.3378-1.7059 | 7.72E-06 |
| *SNORD12B* | 0.2330 | 1.2624 | 1.0735-1.4513 | 0.0156 |
| *SNORD93* | 0.2680 | 1.3073 | 1.1077-1.5069 | 0.0085 |
| *SNORA59B* | -0.1322 | 0.8762 | 0.7359-0.9165 | 0.0349 |
| *SNORD116-2* | -0.2461 | 0.7818 | 0.5385-0.9252 | 0.0275 |
| *SNORA2* | -0.2791 | 0.7565 | 0.5397-0.9032 | 0.0116 |

Abbreviation: HR, hazard ratio.

^a^Values >0.0 and ^b^Values >1.0 indicate that expression is positively associated with poor survival.

^c^Likelihood ratio test *P* value.
