# Supplementary material for: Expression signature of six‐snoRNA serves as novel non‐invasive biomarker for diagnosis and prognosis prediction of renal clear cell carcinoma
Source: J Cell Mol Med. 2020 Jan 14;24(3):2215–28. doi: 10.1111/jcmm.14886 (PMC7011154; doi:10.1111/jcmm.14886)
Supplement: Supplementary file 6 [file JCMM-24-2215-s006.docx]

**Table S5. Univariable Cox regression analysis of the risk score and clinical information for OS in test, validation and entire series**

| **Variables** | **Univariable analysis** | | |
| --- | --- | --- | --- |
|  | **HR** | **95% CI** | ***P* value** |
| **Test series** |  |  |  |
| Risk score (High vs low)^a^ | 3.110 | 2.092-4.624 | **<0.0001** |
| Age (>65 vs ≤65) | 1.435 | 1.009-2.042 | **0.0440** |
| Gender (Male vs female) | 1.042 | 0.722-1.504 | 0.8260 |
| TNM (I/ II/ III/ IV) | 1.972 | 1.683-2.310 | **<0.0001** |
| Fuhrman grade (I+II/ III/ IV) | 2.470 | 1.942-3.142 | **<0.0001** |
| Hemoglobin (Low vs normal level) | 2.219 | 1.470-3.351 | **<0.0001** |
| **Validation series** |  |  |  |
| Risk score (High vs low) ^a^ | 3.439 | 1.809-6.535 | **<0.0001** |
| Age (>65 vs ≤65) | 2.367 | 1.336-4.195 | **0.0030** |
| Gender (Male vs female) | 0.763 | 0.427-1.361 | 0.3600 |
| TNM (I/ II/ III/ IV) | 1.777 | 1.389-2.273 | **<0.0001** |
| Fuhrman grade (I+II/ III/ IV) | 1.984 | 1.338-2.942 | **0.0010** |
| Hemoglobin (Low vs normal level) | 2.610 | 1.275-5.346 | **0.0090** |
| **Entire series** |  |  |  |
| Risk score (High vs low) ^a^ | 3.237 | 2.309-4.537 | **<0.0001** |
| Age (>65 vs ≤65) | 1.658 | 1.229-2.237 | **0.0010** |
| Gender (Male vs female) | 0.953 | 0.699-1.299 | 0.7600 |
| TNM (I/ II/ III/ IV) | 1.886 | 1.653-2.152 | **<0.0001** |
| Fuhrman grade (I+II/ III/ IV) | 2.314 | 1.884-2.841 | **<0.0001** |
| Hemoglobin (Low vs normal level) | 2.332 | 1.632-3.332 | **<0.0001** |

Abbreviation: HR, hazard ratio; 95% CI, 95% confidence interval.

NOTE: Bold, significant values < 0.05.

^a^The 6-snoRNA signature risk score was categorized on the basis of median.
