# Supplementary material for: Expression signature of six‐snoRNA serves as novel non‐invasive biomarker for diagnosis and prognosis prediction of renal clear cell carcinoma
Source: J Cell Mol Med. 2020 Jan 14;24(3):2215–28. doi: 10.1111/jcmm.14886 (PMC7011154; doi:10.1111/jcmm.14886)
Supplement: Supplementary file 7 [file JCMM-24-2215-s007.docx]

**Table S6. Multivariable Cox regression analysis of the risk score and clinical information for OS**

| **Variables** | **Multivariable analysis** | | |
| --- | --- | --- | --- |
|  | **HR** | **95% CI** | ***P* value** |
| **Test series** |  |  |  |
| Risk score (High vs low)^a^ | 2.184 | 1.431-3.336 | **<0.0001** |
| Age (>65 vs ≤65) | 1.487 | 1.029-2.150 | **0.0350** |
| TNM (I/ II/ III/ IV) | 1.529 | 1.265-1.849 | **<0.0001** |
| Fuhrman grade (I+II/ III/ IV) | 1.432 | 1.079-1.900 | **0.0130** |
| Hemoglobin (Low vs normal level) | 1.701 | 1.108-2.610 | **0.0150** |
| **Validation series** |  |  |  |
| Risk score (High vs low) ^a^ | 3.873 | 1.825-8.217 | **<0.0001** |
| Age (>65 vs ≤65) | 1.821 | 0.953-3.476 | 0.0690 |
| TNM (I/ II/ III/ IV) | 1.645 | 1.214-2.229 | **0.0010** |
| Fuhrman grade (I+II/ III/ IV) | 1.369 | 1.093-2.013 | **0.0210** |
| Hemoglobin (Low vs normal level) | 1.566 | 1.086-2.846 | **0.0241** |
| **Entire series** |  |  |  |
| Risk score (High vs low) ^a^ | 2.540 | 1.766-3.653 | **<0.0001** |
| Age (>65 vs ≤65) | 1.567 | 1.143-2.149 | **0.0050** |
| TNM (I/ II/ III/ IV) | 1.535 | 1.311-1.797 | **<0.0001** |
| Fuhrman grade (I+II/ III/ IV) | 1.392 | 1.097-1.766 | **0.0070** |
| Hemoglobin (Low vs normal level) | 1.617 | 1.115-2.346 | **0.0110** |

Abbreviation: HR, hazard ratio; 95% CI, 95% confidence interval.

NOTE: Bold, significant values < 0.05.

^a^The 6-snoRNA signature risk score was categorized on the basis of median.
