# Supplementary material for: Expression signature of six‐snoRNA serves as novel non‐invasive biomarker for diagnosis and prognosis prediction of renal clear cell carcinoma
Source: J Cell Mol Med. 2020 Jan 14;24(3):2215–28. doi: 10.1111/jcmm.14886 (PMC7011154; doi:10.1111/jcmm.14886)
Supplement: Supplementary file 8 [file JCMM-24-2215-s008.docx]

**Table S7. Univariable Cox regression analysis of the risk score and clinical information for RFS**

| **Variables** | **Univariable analysis** | | |
| --- | --- | --- | --- |
|  | **HR** | **95% CI** | ***P* value** |
| **Entire series** |  |  |  |
| Risk score (High vs low) ^a^ | 1.212 | 1.142-1.282 | **<0.0001** |
| Age (>65 vs ≤65) | 1.506 | 1.051-1.961 | **0.0020** |
| Gender (Male vs female) | 1.122 | 0.766-1.478 | 0.4150 |
| TNM (I/ II/ III/ IV) | 2.192 | 1.899-2.485 | **<0.0001** |
| Fuhrman grade (I+II/ III/ IV) | 2.342 | 1.857-2.827 | **<0.0001** |
| Hemoglobin (Low vs normal level) | 1.841 | 1.208-2.474 | **<0.0001** |

Abbreviation: HR, hazard ratio; 95% CI, 95% confidence interval.

NOTE: Bold, significant values < 0.05.

^a^The 6-snoRNA signature risk score was categorized on the basis of median.
