# Supplementary material for: Expression signature of six‐snoRNA serves as novel non‐invasive biomarker for diagnosis and prognosis prediction of renal clear cell carcinoma
Source: J Cell Mol Med. 2020 Jan 14;24(3):2215–28. doi: 10.1111/jcmm.14886 (PMC7011154; doi:10.1111/jcmm.14886)
Supplement: Supplementary file 9 [file JCMM-24-2215-s009.docx]

**Table S8. Multivariable Cox regression analysis of the risk score and clinical information for RFS**

| **Variables** | **Multivariable analysis** | | |
| --- | --- | --- | --- |
|  | **HR** | **95% CI** | ***P* value** |
| **Entire series** |  |  |  |
| Risk score (High vs low) ^a^ | 1.096 | 1.031-1.161 | **0.0020** |
| Age (>65 vs ≤65) | 1.524 | 1.028-2.020 | **0.0030** |
| TNM (I/ II/ III/ IV) | 1.819 | 1.531-2.107 | **<0.0001** |
| Fuhrman grade (I+II/ III/ IV) | 1.384 | 1.058-1.710 | **0.0030** |
| Hemoglobin (Low vs normal level) | 1.356 | 1.072-1.640 | **0.0410** |

Abbreviation: HR, hazard ratio; 95% CI, 95% confidence interval.

NOTE: Bold, significant values < 0.05.

^a^The 6-snoRNA signature risk score was categorized on the basis of median.
