# Supplementary material for: Expression signature of six‐snoRNA serves as novel non‐invasive biomarker for diagnosis and prognosis prediction of renal clear cell carcinoma
Source: J Cell Mol Med. 2020 Jan 14;24(3):2215–28. doi: 10.1111/jcmm.14886 (PMC7011154; doi:10.1111/jcmm.14886)
Supplement: Supplementary file 10 [file JCMM-24-2215-s010.docx]

**Table S9. Correlation between risk score and clinicopathologic features of ccRCC patients**

| **Characteristics** | **N** | **Risk score level** | | |
| --- | --- | --- | --- | --- |
|  |  | **Low** | **High** | ***P* value^a^** |
| Gender |  |  |  | 1.0000 |
| Male | 22 | 11 | 11 |  |
| Female | 10 | 5 | 5 |  |
| Age(years) |  |  |  | 0.2380 |
| >65 | 9 | 6 | 3 |  |
| ≤65 | 23 | 10 | 13 |  |
| TNM stage |  |  |  | **0.0030** |
| I and II | 19 | 14 | 5 |  |
| III and IV | 8 | 1 | 7 |  |
| Fuhrman grade |  |  |  | **0.0020** |
| I and II | 21 | 15 | 6 |  |
| III and IV | 6 | 0 | 6 |  |
| White blood cell count |  |  |  | 0.1890 |
| Normal | 25 | 13 | 12 |  |
| Non-normal | 2 | 2 | 0 |  |
| Red blood cell count |  |  |  | 0.8090 |
| Normal | 23 | 13 | 10 |  |
| Non-normal | 4 | 2 | 2 |  |
| Platelet count |  |  |  | 0.8250 |
| Normal | 22 | 12 | 10 |  |
| Non-normal | 5 | 3 | 2 |  |

NOTE: Bold, significant values < 0.05.

^a^*P* values were calculated by Fisher exact test.
