# Supplementary material for: Expression signature of six‐snoRNA serves as novel non‐invasive biomarker for diagnosis and prognosis prediction of renal clear cell carcinoma
Source: J Cell Mol Med. 2020 Jan 14;24(3):2215–28. doi: 10.1111/jcmm.14886 (PMC7011154; doi:10.1111/jcmm.14886)
Supplement: Supplementary file 11 [file JCMM-24-2215-s011.docx]

**Table S10. snoRNPs genes related with high-risk group and low-risk group**

| highrisk_snoRNPs | | | | lowrisk_snoRNPs | | |
| --- | --- | --- | --- | --- | --- | --- |
| ADAR | GTF2F1 | PTBP1 | TCF7L2 | ACIN1 | HNRNPA2B1 | SLBP |
| AIFM1 | HEY1 | RAD21 | TFAP2A | ADAR | HNRNPC | SLTM |
| ALYREF | HNF4A | RANGAP1 | TIA1 | AUH | HNRNPK | SMAD3 |
| BATF | HNRNPA1 | RBFOX2 | TIAL1 | BCCIP | HNRNPM | SMARCA4 |
| BCCIP | HNRNPA2B1 | RBM22 | TRA2A | BCL11A | HNRNPU | SMARCB1 |
| BCL11A | HNRNPC | RBM27 | U2AF1 | BCL3 | HNRNPUL1 | SMARCC1 |
| BCL3 | HNRNPK | RBM39 | U2AF2 | BDP1 | IGF2BP1 | SMARCC2 |
| BUD13 | HNRNPM | RBM47 | UPF1 | BUD13 | IGF2BP2 | SMNDC1 |
| CDX2 | HNRNPU | RBM5 | VIM | CDX2 | IGF2BP3 | SP1 |
| CEBPB | HNRNPUL1 | RBM6 | WTAP | CEBPB | ILF3 | SRSF1 |
| CPSF6 | IGF2BP1 | REST | XRN2 | CSTF2T | JUN | SRSF10 |
| CSTF2T | IGF2BP2 | RNF219 | YTHDC1 | DDX3X | KHDRBS1 | SRSF3 |
| CTCF | IGF2BP3 | RTCB | YTHDF1 | DDX54 | KHSRP | SRSF7 |
| DDX3X | IRF4 | RXRA | YWHAG | DGCR8 | LARP7 | SRSF9 |
| DDX42 | JUN | SAFB2 | YY1 | DHX9 | LIN28A | STAT1 |
| DDX54 | JUND | SBDS | ZBTB33 | DKC1 | LIN28B | TAF1 |
| DGCR8 | KHDRBS1 | SETDB1 | ZC3H7B | E2F1 | LSM11 | TAF15 |
| DHX9 | LARP7 | SIX5 | ZNF184 | E2F4 | MAX | TARDBP |
| DKC1 | LIN28A | SLTM | ZNF263 | E2F6 | MBNL2 | TCF12 |
| E2F1 | LIN28B | SMAD3 |  | EBF1 | METTL14 | TCF7L2 |
| E2F4 | LSM11 | SMAD4 |  | EIF4A3 | METTL3 | TNRC6A |
| E2F6 | MAX | SMARCA4 |  | EIF4G2 | MYC | TRA2A |
| EBF1 | MBNL1 | SMARCB1 |  | ELAVL1 | NANOG | TROVE2 |
| EIF4A3 | MBNL2 | SMARCC1 |  | EP300 | NCBP3 | U2AF1 |
| EIF4G2 | MED12 | SMARCC2 |  | ERG | NELFE | U2AF2 |
| ELAVL1 | METTL14 | SMNDC1 |  | ESRRA | NFYA | UPF1 |
| ELAVL3 | METTL3 | SND1 |  | ETS1 | NFYB | WTAP |
| EP300 | MSI2 | SP1 |  | FBL | NONO | XRN2 |
| ERG | MYC | SPI1 |  | FMR1 | NOP58 | YWHAG |
| ETS1 | NANOG | SREBF1 |  | FOS | NUMA1 | YY1 |
| EWSR1 | NCBP3 | SRSF1 |  | FOSL2 | PBX3 | ZBTB33 |
| FAM120A | NELFE | SRSF10 |  | FTO | PCBP2 | ZNF184 |
| FMR1 | NONO | SRSF3 |  | FUS | POU2F2 | ZNF263 |
| FOSL2 | NPM1 | SRSF7 |  | FXR1 | PRPF8 |  |
| FOXP2 | NRF1 | SRSF9 |  | GATA6 | PTBP1 |  |
| FUS | NUMA1 | STAT1 |  | GNL3 | RBFOX2 |  |
| FXR1 | PBX3 | STAT2 |  | GTF2B | RBM10 |  |
| FXR2 | PCBP2 | TAF1 |  | GTF2F1 | RBM27 |  |
| GATA6 | POU2F2 | TAF15 |  | HEY1 | REST |  |
| GNL3 | PPARGC1A | TARDBP |  | HNF4A | RNF219 |  |
| GTF2B | PRPF8 | TCF12 |  | HNRNPA1 | SF3B4 |  |
